# Technical and tactical performance of major Asian professional football leagues: a comparative analysis of 14,115 matches across six seasons

**DOI:** 10.5114/biolsport.2026.159568

**Published:** 2026-04-13

**Authors:** Liangzhu Feng, Yanlong Chen, Zhe Cheng, Hongyou Liu

**Affiliations:** 1 Research Center of Sports Performance Analysis, School of Physical Education & Sports Science, South China Normal University, Guangzhou, China; 2 Guangdong University of Foreign Studies, Guangzhou, China; 3 Zhujiang Middle school, Guangzhou, China

**Keywords:** Match analysis, Cross-league comparison, Contextual variables, Asian football, Sport performance

## Abstract

This study aimed to identify cross-league differences in technical and tactical performance across Asian professional football leagues. Statistics of 14,115 matches from the 2015–2020 seasons of the Chinese Super League (CSL), J League 1 (J1), K League 1 (K1), Saudi Professional League (SPL), Qatar Stars League (QSL), and UAE Arabian Gulf League (AGL) were collected and analysed. Generalised mixed linear modelling was used to estimate mean league differences in 57 match performance-related parameters while controlling for game result, match location, and team and opponent strength. Results showed that differences in offensive outcome and performance-related parameters were generally trivial across leagues (ES ≤ 0.2). In contrast, possession- and progression-related parameters exhibited substantial between-league differences, with J1 demonstrating higher passing frequency and ball progression parameters than the other leagues (ES = 0.36–0.91). Conversely, SPL, CSL, QSL, and AGL recorded higher frequencies of defensive and disciplinary actions, including fouls and yellow cards, compared with J1 and K1 (ES = 0.46–1.33). Hence, it is concluded that technical and tactical performance is generally similar across major Asian professional football leagues, but notable stylistic differences remain. J1 tends to exhibit a possession-oriented and structured playing style, whereas SPL, CSL, QSL, and AGL are characterised by greater physical intensity and more confrontational play. These insights may inform performance analysis and coaching strategies across Asian professional football.

## INTRODUCTION

Comparison of the match performance across leagues can reveal differences in tactical styles, technical preferences, and match intensity, clarifying “league style” as a macro-level contextual factor [1] and providing evidence to guide adaptation to unfamiliar opponents and to evaluate competitive gaps between leagues [2–4].

Early cross-league studies focused on physical and technical performance of players. Rienzi et al [5] reported that South American players covered less distance than European players, reflecting cultural and tempo-related differences. Bloomfield et al. [6] analysed elite players across four European leagues during the 2001–2002 season, showing systematic differences in age, stature, body mass, and BMI among leagues, which highlighted the influence of league context on player characteristics. Dellal et al. [7] compared the English Premier League (EPL) and Spanish La Liga during the 2006–2007 season, finding that EPL players performed more high-intensity running and sprints, whereas La Liga players achieved higher passing accuracy and longer possession times. Collectively, these studies confirm league-specific characteristics shaped by cultural and competitive contexts.

Later on, research on team offensive strategies emphasised how offensive efficiency and tactical choices vary across leagues. Sarmento et al. [8] analysed 1,694 offensive sequences in 68 matches from La Liga, Serie A, Bundesliga, EPL, and the UEFA Champions League, classifying attacks as combinative, fast, direct, or counteroffensive. Counterattacks and fast attacks were associated with approximately 40% higher success rates than other offensive patterns, and effectiveness differed across leagues, with Spanish, Italian, and English matches outperforming UEFA Champions League games. González-Rodenas [9] confirmed that counterattacks and fast attacks are generally more effective, while direct attacks are less efficient despite frequent use in both the EPL and La Liga during the 2017–2018 season (n = 380 matches).

Methodological advances have enabled multi-dimensional crossleague analyses that integrate tactical and contextual factors. Yi et al. [10] incorporated contextual variables, i.e., match location, match type, game result, and opponent strength, when comparing technical performance of players from Bundesliga, La Liga, Ligue 1, Premier League, and Serie A players during 2009–2018 seasons in the UEFA Champions League. Building on this, Aranda-Malavés et al. [11] incorporated tactical dimensions, including starting zones, width of possession, and pressing intensity, alongside contextual variables to explain variations in offensive behaviours across leagues.

Despite these advances, most cross-league studies have concentrated on Europe’s “big five” leagues or UEFA Champions League [3, 10, 12]. By contrast, research on Asian professional football has primarily examined single leagues in isolation, focusing on aspects such as physical and technical demands [13], passing networks [14], or contextual influences [15]. However, these studies have not systematically addressed cross-league or cross-culture comparisons, leaving broader patterns of tactical and technical performance across Asia largely unexplored. This gap is particularly evident given that, in recent decades, Asian professional football has undergone rapid professionalisation, characterised by increased financial investment, greater involvement of foreign players and coaches, and rising competitive standards [16–18]. At the same time, substantial cross-league differences in regulatory frameworks and culturally influenced playing styles suggest the potential for distinct league-specific technical and tactical performance profiles.

Based on these considerations, this study examined six major Asian professional football leagues representing diverse and well-established professional football contexts. League selection was based on competitive level, professionalisation, and regular participation in AFC continental competitions. A large dataset comprising 14,115 matches across six seasons was analysed using generalised mixed linear modelling, while controlling for match outcome, match location, team strength, and opponent strength, to investigate cross-league variations in technical and tactical match performance. The aim was to provide a more comprehensive assessment of similarities and differences across Asian professional football leagues.

## MATERIALS AND METHODS

### Sample

The sample of this study was composed of the match performance statistics of 14,115 matches from the 2015 to 2020 seasons across six Asian professional football leagues: the Chinese Super League (CSL, n = 2404), Japan’s J1 League (J1, n = 3375), Korea’s K1 League (K1, n = 2201), the Saudi Professional League (SPL, n = 2367), the Qatar Stars League (QSL, n = 1778), and the UAE Arabian Gulf League (AGL, n = 1990). Original data were obtained from WyScout, a professional football data company whose datasets have been widely applied in training and performance analysis in international football, and whose reliability has been previously verified [19–21]. The dataset included technical, tactical, and contextual information for each team in each match. Ethical approval for this study was granted by the local university ethics committee.

### Variables

In line with prior literature, contextual variables such as league type, match location (home/away), game result (win/draw/loss), team strength (final season ranking: 1–20), and opponent strength (final season ranking: 1–20) were selected as independent variables. Technical and tactical performance parameters of each team in each match were chosen as dependent variables. The grouping and operational definitions of these variables are presented in Table 1.

**TABLE 1:** Selected Offensive outcome, Passing, Defensive outcome and Set-Piece performance-related parameters (dependent variables).

***Offensive outcome and performance-related parameters (unit): operational definition***

**GoalScored:** number of goals scored by a team.
**ExpectedGoals:** sum of probabilities for each shot to result in a goal, calculated using an xG model.
**Shot:** attempts to score a goal, using any legal part of the body, on or off target.
**ShotAcc (%):** proportion of shots on target out of total shots.
**ShotonTarget:** number of shots on target.
**ShotOutsideTargetAcc (%):**proportion of shots on target from outside the penalty area.
**AverageShotDistance:** average distance from goal for all shots.
**PositionalAtk:** number of attacks organised from structured play.
**PositionalAtkSuss (%):** proportion of positional attacks that result in at least one shot.
**CounterAtk:** counter-attacks initiated immediately after regaining possession.
**CounterAtkwithShot (%):** proportion of counter-attacks that result in at least one shot.
**EnterPenaltyArea:** number of entries into the opponent’s penalty area with the ball.
**DribbleIntoPenaltyArea:** number of times players carry the ball into the opponent’s penalty area.
**CrossIntoPenaltyArea:** number of crosses delivered into the opponent’s penalty area.
**TouchInPenaltyArea:** total touches inside the opponent’s penalty area.
**Loss:** number of times the team loses possession.
**Offsides:** number of offside offenses committed by the team.

***Passing performance-related parameters (unit): operational definition***

**Pass:** intentional passes from one player to another.
**PassAcc (%):** successful passes as a proportion of total passes.
**LongPassPropotion (%):** long passes as a proportion of total passes.
**FwdPass:** forward passes into the opponent’s half.
**FwdPassAcc (%):** successful forward passes as a proportion of total forward passes.
**BackPass:** number of passes directed toward own half.
**BackPassAcc (%):** successful backward passes as a proportion of total backward passes.
**LateralPass:** lateral passes across the pitch.
**LateralPassAcc (%):** successful lateral passes as a proportion of total lateral passes.
**LongPass:** total number of long passes.
**LongPassAcc (%):** successful long passes as a proportion of total long passes.
**PasstoAT:** passes into offensive third.
**PasstoATAcc (%):** successful passes into offensive third as a proportion of total passes into that area.
**ProgresivePasst:** number of progressive passes advancing the ball significantly toward opponent’s goal.
**ProgresivePasstAcc (%):** successful progressive passes as a proportion of total progressive passes.
**Cross:** crosses into dangerous areas.
**CrossAcc (%):** successful crosses as a proportion of total crosses.
**PassperMin:** average passes per minute of match time.
**PassesperPossession:** average passes per possession.
**AveragePassLength:** average distance of passes.
**Pass/DefensiveActions:** ratio of passes to defensive actions.

***Defensive outcome and Set-Piece performance-related parameters (unit): operational definition***

**Recovery:** number of times the team regains possession.
**Challenge:** actions in which two players compete for a ball not under control of either.
**AerialDuel:** duels contested in the air.
**Tackle:** attempts to dispossess an opponent in control of the ball.
**TackleSuss (%):** successful tackles as a proportion of total tackles.
**Interception:** number of times the team intercepts opponent passes.
**Clearance:** number of times the team clears the ball from defensive area.
**Foul:** number of fouls committed.
**Yellowcard (%):**proportion of yellow cards relative to total disciplinary actions.
**Redcard (%):**proportion of red cards relative to total disciplinary actions.
**SetPiece:** set-piece situations (corners, free kicks, penalties).
**SetPieceSuss (%):** proportion of set-pieces leading to shots.
**Corner:** number of corner kicks awarded.
**CornerSuss (%):** proportion of corners leading to shots.
**Freekick:** number of free kicks awarded.
**FreekickSuss (%):** proportion of free kicks leading to shots.
**Penalty:** number of penalties awarded.
**PenaltyConvert (%):** goals scored from penalties as a proportion of total penalties.
**GoalKick:** number of goal kicks taken.

Note: parameters without unit are counts.

### Statistical analysis

The generalised mixed linear modelling was realised with Proc Glimmix in the University Edition of Statistical Analysis System (SAS^®^ Studio, version 3.6). Separate Poisson (for count variables) and Beta (for proportion variables) regressions were run in the model, taking the value of each technical and tactical performance-related parameter as the dependent variable. A random effect for team identity was included to account for repeated measurements on the same teams across multiple matches. The fixed effects estimated the effects of five predictor variables: league type, game result, match location, team strength, and opponent strength.

League type, game result, and match location were included as nominal variables. League type was coded with six levels (1 = CSL, 2 = J1, 3 = K1, 4 = SPL, 5 = QSL, 6 = AGL). Game result had three levels (3 = win, 1 = draw, 0 = loss), and match location had two levels (1 = home, 2 = away). The effect of team strength and opponent strength was estimated by including the difference in the log of the end-of-season ranks as a predictor [22].

The established models estimated the mean values of each technical and tactical performance parameter for teams across the six leagues, while controlling for the contextual effects of game result, match location, team strength, and opponent strength. Observed magnitudes and their confidence limits were expressed in standardised effect sizes (ES), whereby the estimated mean difference was divided by the observed between-match standard deviation derived from the mixed model. The ES was then evaluated qualitatively with the following scale: < 0.2 trivial, 0.2–0.6 small, 0.6–1.2 moderate, 1.2–2.0 large, and > 2.0 very large. Effects were deemed clear if the 99% confidence interval did not simultaneously include both −0.2 and 0.2. Clear effects were reported with a qualitative likelihood that the true change was either substantial or trivial (whichever probability was greater) using the following scale: < 0.5% most unlikely, 0.5–5% very unlikely, 5–25% unlikely, 25–75% possibly, 75–95% likely, 95–99.5% very likely, > 99.5% most likely [23]. To minimize statistical error, only effects with a probability greater than 95% were considered in the analysis and discussion.

## RESULTS

### Offensive outcome and performance of teams across different professional leagues

**FIG. 1:**
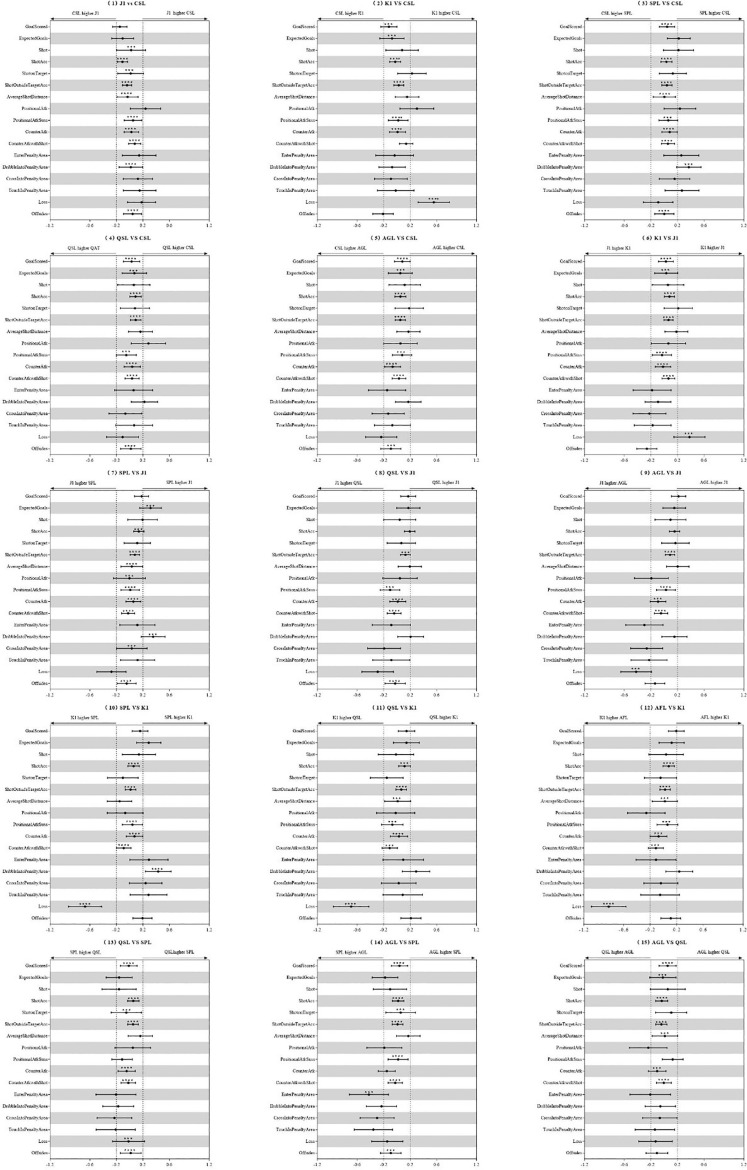
Standardised mean differences of offensive outcome and performance-related parameters between the leagues estimated from the generalised mixed linear modelling. Filled dots are the standardised mean differences (ES values). Error bars are the 99% confidence intervals. Number of asterisks indicate the likelihood for the magnitude of the true change as follows: 3-very likely; 4-most likely.

Descriptive statistics of offensive outcome and performance across the six leagues are presented in Table 2, and comparative trends estimated from the generalised mixed models are shown in Figure 1. Overall, differences in most offensive parameters were trivial across all leagues. Loss was higher in K1 than in J1, SPL, and AGL, and higher in J1 than in AGL. Additionally, DribbleIntoPenaltyArea was higher in SPL than in J1 and K1, while EnterPenaltyArea was higher in SPL than in AGL.

**TABLE 2:** Offensive outcome and performance parameters across major Asian professional football leagues (mean ± SD)

	CSL	J1	K1	SPL	QSL	AGL
GoalScored	1.5 ± 1.3	1.3 ± 1.2	1.3 ± 1.2	1.5 ± 1.2	1.5 ± 1.4	1.6 ± 1.3
ExpectedGoals	1.5 ± 0.8	1.4 ± 0.8	1.4 ± 0.8	1.5 ± 0.9	1.5 ± 1.0	1.5 ± 0.9
Shot	12.0 ± 4.7	12.1 ± 4.5	12.1 ± 4.5	12.2 ± 4.5	12.2 ± 5.0	12.4 ± 5.0
ShotAcc (%)	37 ± 17	35 ± 16	36 ± 16	38 ± 16	39 ± 16	38 ± 16
ShotonTarget	5.3 ± 2.7	5.3 ± 2.6	5.8 ± 2.9	5.3 ± 2.7	5.3 ± 2.7	5.7 ± 2.9
ShotOutsideTargetAcc (%)	27 ± 23	26 ± 23	28 ± 22	28 ± 23	29 ± 23	28 ± 22
AverageShotDistance	19.0 ± 3.4	18.9 ± 3.1	19.5 ± 3.7	19.1 ± 3.2	19.4 ± 3.6	19.6 ± 3.3
PositionalAtk	28.2 ± 8.7	30.5 ± 8.9	30.6 ± 9.2	29.5 ± 9.7	30.5 ± 10.2	28.7 ± 9.4
PositionalAtkSuss (%)	22.6 ± 9.4	23.0 ± 9.0	22.7 ± 8.9	22.5 ± 9.2	22.0 ± 9.0	23.2 ± 9.6
CounterAtk	2.8 ± 3.0	2.9 ± 2.6	2.8 ± 2.7	3.0 ± 2.5	2.9 ± 2.8	2.6 ± 2.6
CounterAtkwithShot (%)	31 ± 32	33 ± 33	36 ± 34	32 ± 32	32 ± 33	32 ± 34
EnterPenaltyArea	22.5 ± 8.2	24.2 ± 8.4	22.4 ± 8.2	24.1 ± 9.1	23.2 ± 9.4	21.7 ± 8.8
DribbleIntoPenaltyArea	2.0 ± 1.7	2.1 ± 1.8	1.8 ± 1.6	2.6 ± 2.0	2.5 ± 2.1	2.3 ± 1.9
CrossIntoPenaltyArea	8.8 ± 4.8	9.3 ± 4.6	8.6 ± 4.8	9.2 ± 5.1	8.3 ± 4.7	8.2 ± 4.8
TouchInPenaltyArea	14.9 ± 6.8	16.2 ± 7.2	14.8 ± 6.6	16.2 ± 7.6	15.8 ± 8.2	14.6 ± 7.0
Loss	103 ± 16	107 ± 17	113 ± 18	102 ± 15	101 ± 15	100 ± 15
Offsides	1.9 ± 1.6	2.0 ± 1.7	1.6 ± 1.5	1.9 ± 1.6	1.9 ± 1.7	1.8 ± 1.6

### Passing performance of teams across different professional leagues

**FIG. 2:**
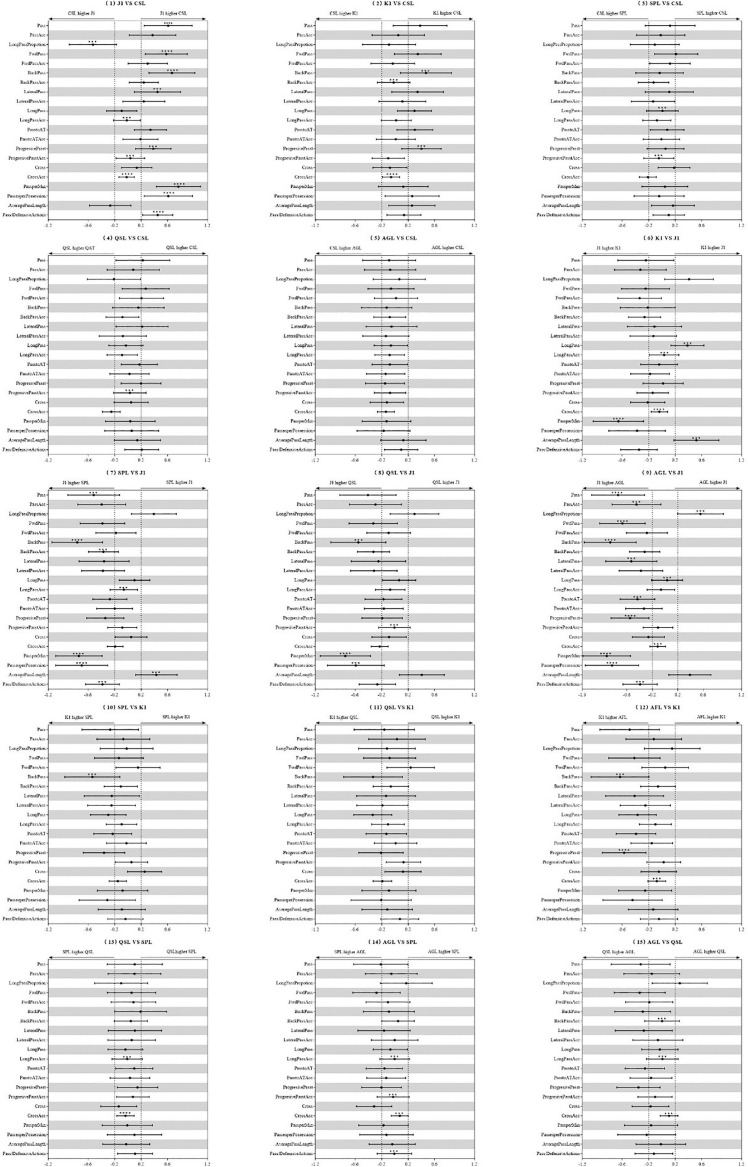
Standardised mean differences of passing performance-related parameters between the leagues estimated from the generalised mixed linear modelling. Filled dots are the standardised mean differences (ES values). Error bars are the 99% confidence intervals. Number of asterisks indicate the likelihood for the magnitude of the true change as follows: 3-very likely; 4-most likely.

Descriptive statistics of passing performance across the six leagues are presented in Table 3, and standardised effect sizes from the generalised mixed models are displayed in Figure 2. In J1, Pass, FwdPass, BackPass, LateralPass, and ProgressivePass were higher than those in the other leagues. PassesPerMin, PassesPerPossession, and Pass/DefensiveActions were also higher in J1 compared with the other leagues. In contrast, LongPass and LongPassProportion were higher in CSL, K1, SPL, and AGL than in J1.

**TABLE 3:** Passing performance parameters across major Asian professional football leagues (mean ± SD)

	CSL	J1	K1	SPL	QSL	AGL
Pass	372 ± 99	449 ± 129	408 ± 99	387 ± 104	397 ± 108	370 ± 115
PassAcc(%)	80.8 ± 5.2	83.2 ± 5.3	81.0 ± 5.0	80.9 ± 5.0	81.4 ± 5.1	80.6 ± 6.0
LongPassPropotion(%)	13.0 ± 4.1	10.8 ± 4.2	12.6 ± 3.9	12.5 ± 4.0	12.2 ± 3.9	13.3 ± 4.7
FwdPass	135 ± 27	155 ± 34	144 ± 26	141 ± 28	142 ± 29	135 ± 32
FwdPassAcc(%)	72.9 ± 6.9	75.5 ± 7.1	72.7 ± 6.8	73.7 ± 6.9	74.4 ± 7.2	73.1 ± 7.7
BackPass	51 ± 16	66 ± 23	59 ± 18	51 ± 17	54 ± 18	50 ± 19
BackPassAcc(%)	91.2 ± 5.0	92.6 ± 4.6	91.1 ± 4.8	91.2 ± 5.3	91.0 ± 5.1	90.8 ± 5.7
LateralPass	129 ± 48	156 ± 58	144 ± 51	135 ± 50	141 ± 53	130 ± 54
LateralPassAcc(%)	85.5 ± 5.5	87.1 ± 5.3	86.0 ± 5.2	85.0 ± 5.5	85.3 ± 5.3	84.9 ± 6.2
LongPass	46 ± 10	45 ± 11	49 ± 11	45 ± 10	45 ± 10	45 ± 11
LongPassAcc(%)	54.9 ± 9.1	55.0 ± 9.0	55.1 ± 8.8	54.2 ± 9.4	54.5 ± 9.6	54.1 ± 9.3
PasstoAT	56 ± 15	62 ± 17	60 ± 16	57 ± 16	58 ± 18	56 ± 17
PasstoATAcc(%)	68.5 ± 9.7	70.9 ± 9.8	68.4 ± 9.5	68.2 ± 10.0	68.8 ± 10.4	67.3 ± 10.5
ProgresivePasst	74 ± 17	82 ± 18	80 ± 17	74 ± 16	77 ± 19	72 ± 18
ProgresivePasstAcc(%)	76.5 ± 8.6	77.1 ± 8.5	75.4 ± 8.9	75.8 ± 9.4	76.5 ± 9.8	75.9 ± 9.7
Cross	14.9 ± 6.9	15.7 ± 6.6	14.6 ± 6.9	15.6 ± 7.6	14.9 ± 7.4	14.1 ± 7.1
CrossAcc(%)	34 ± 14	34 ± 14	33 ± 15	31 ± 14	30 ± 14	32 ± 15
PassperMin	15.4 ± 1.6	16.8 ± 1.8	15.7 ± 1.4	15.6 ± 1.4	15.6 ± 1.4	15.3 ± 1.7
PassesperPossession	3.5 ± 1.0	4.3 ± 1.3	3.8 ± 1.0	3.5 ± 1.0	3.6 ± 1.0	3.4 ± 1.1
AveragePassLength	20.2 ± 1.6	19.7 ± 1.9	20.6 ± 1.6	20.3 ± 1.5	20.4 ± 1.5	20.5 ± 1.8
Pass/DefensiveActions	9.5 ± 4.5	11.9 ± 6.0	10.3 ± 4.6	10.4 ± 5.1	10.8 ± 5.7	10.0 ± 5.2

In CSL, LongPassProportion was higher than in J1. K1 recorded higher BackPass and ProgressivePass than CSL and AGL, higher BackPass than SPL, and higher LongPass and AveragePassLength than J1. SPL showed higher AveragePassLength than J1, while AGL exhibited a higher LongPassProportion than J1.

### Defensive outcome and set-piece performance of teams across different professional leagues

**FIG. 3:**
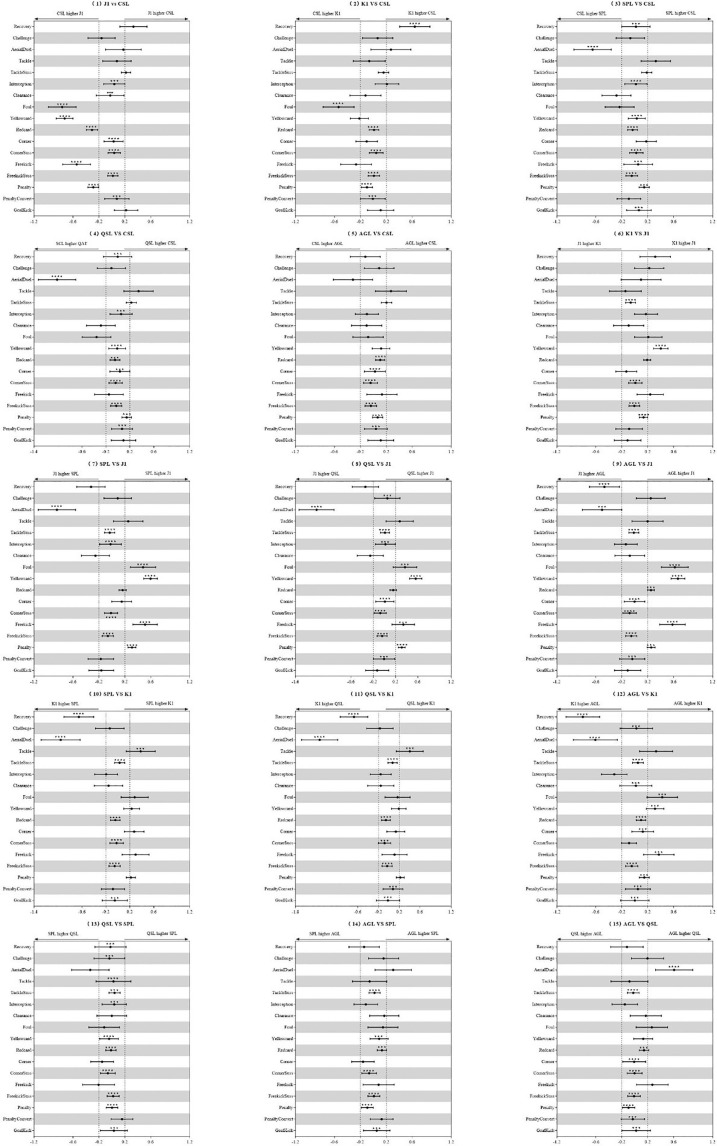
Standardised mean differences of defensive outcome and set-piece performance-related parameters between the leagues estimated from the generalised mixed linear modelling. Filled dots are the standardised mean differences (ES values). Error bars are the 99% confidence intervals. Number of asterisks indicate the likelihood for the magnitude of the true change as follows: 3-very likely; 4-most likely.

Descriptive statistics of defensive outcome and set-piece performance are presented in Table 4, with standardised effect sizes displayed in Figure 3. Overall, SPL, QSL, AGL, and CSL recorded higher Foul, Yellowcard, Freekick, and Penalty than J1 and K1, whereas J1 and K1 demonstrated higher AerialDuel than SPL, QSL, and AGL. Recovery in K1 was higher than in AGL and CSL, while Yellowcard was higher than in J1. Additionally, Tackle was higher in SPL and QSL than in K1, and AerialDuel was higher in AGL than in QSL.

**TABLE 4:** Defensive outcome and set-piece performance parameters major Asian professional football leagues (mean ± SD)

	CSL	J1	K1	SPL	QSL	AGL
Recovery	75 ± 12	80 ± 12	84 ± 14	75 ± 12	75 ± 12	74 ± 12
Challenge	210 ± 34	203 ± 33	211 ± 37	206 ± 32	204 ± 34	211 ± 34
AerialDuel	41 ± 13	43 ± 15	45 ± 14	33 ± 11	30 ± 10	37 ± 12
Tackle	5.4 ± 3.1	5.7 ± 3.2	5.2 ± 3.0	6.2 ± 3.7	6.5 ± 3.6	6.3 ± 3.3
TackleSuss(%)	42 ± 26	48 ± 26	46 ± 27	47 ± 24	48 ± 24	47 ± 23
Interception	43 ± 11	43 ± 11	45 ± 11	44 ± 11	43 ± 11	42 ± 11
Clearance	18.4 ± 8.3	18.3 ± 8.7	17.6 ± 8.4	17.2 ± 8.4	16.6 ± 8.2	17.7 ± 8.4
Foul	15.2 ± 4.5	12.2 ± 3.9	13.1 ± 4.1	14.3 ± 4.2	13.7 ± 4.4	15.0 ± 4.4
Yellowcard	1.9 ± 1.3	1.2 ± 1.1	1.7 ± 1.2	2.0 ± 1.3	2.0 ± 1.3	2.1 ± 1.3
Redcard	0.10 ± 0.32	0.04 ± 0.20	0.10 ± 0.32	0.10 ± 0.32	0.09 ± 0.30	0.14 ± 0.38
Corner	4.8 ± 2.7	4.9 ± 2.6	4.5 ± 2.5	5.0 ± 2.8	4.8 ± 2.8	4.9 ± 2.8
CornerSuss(%)	28 ± 25	29 ± 24	29 ± 25	28 ± 24	27 ± 24	27 ± 25
Freekick	3.4 ± 2.1	2.4 ± 1.7	2.9 ± 1.8	3.2 ± 2.0	3.0 ± 1.9	3.6 ± 2.1
FreekickSuss(%)	22 ± 27	22 ± 31	22 ± 29	20 ± 27	21 ± 28	21 ± 26
Penalty	0.19 ± 0.41	0.10 ± 0.31	0.15 ± 0.37	0.24 ± 0.48	0.25 ± 0.49	0.22 ± 0.47
PenaltyConvert	79 ± 40	82 ± 38	79 ± 40	76 ± 42	83 ± 37	81 ± 39
GoalKick	8.1 ± 3.4	8.8 ± 3.4	8.5 ± 3.3	8.6 ± 3.4	8.5 ± 3.6	8.5 ± 3.6

## DISCUSSION

This study aimed to quantify cross-league differences in offensive outcome, passing performance, defensive outcome and set-piece performance across six major Asian professional leagues (CSL, J1, K1, SPL, QSL, AGL) over six seasons, while controlling for game result, match location, team strength, and opponent strength. Contrary to common stereotypes, between-league gaps in offensive outcome and performance were small, whereas style remained differentiated— most notably in passing behaviour and defensive/disciplinary profiles.

### Offensive outcome and performance

Between-league differences in offensive outcome and performance were mostly trivial, with no substantial variation in core parameters such as goals scored, shooting accuracy, or shot distance. This suggests that offensive efficiency and overall offensive quality have become increasingly homogeneous across Asian leagues. This contrasts with evidence from European contexts, where clear league-specific offensive patterns remain evident—for example, the Premier League’s direct and high-intensity transitions versus La Liga’s possessionoriented structures [7, 8]. The convergence in Asia may be driven by the global dissemination of tactical frameworks, the widespread presence of foreign coaches and players, and the adoption of standardised training methodologies [12, 24]. It may also reflect the present study’s longitudinal, context-controlled design, which reduces situational and sampling bias [10, 23].

Despite this convergence, notable regional contrasts emerged. K1 recorded significantly higher ball losses than J1, SPL, and AGL, indicating a greater emphasis on high-risk, high-tempo offensive transitions. This pattern is consistent with the league’s reputation for intense pressing and quick turnovers [13]. In contrast, SPL teams demonstrated superior dribbling into the penalty area and more entries into the penalty area than J1, K1, and AGL, suggesting a more aggressive, penetration-oriented offensive approach. The SPL’s emphasis on direct dribble penetration likely reflects its recent influx of elite offensive players and tactical modernisation supported by increased financial investment [11]. AGL, meanwhile, showed fewer penalty area entries, implying a more conservative and controlled offensive strategy. These findings collectively indicate that, while offensive outcomes are relatively uniform, the tactical expression of attack varies according to league context, competition tempo, and structural influences such as foreign player quotas and climatic conditions [3, 25–27].

### Passing performance

Differences in technical passing accuracy were small across leagues, consistent with cross-competition evidence showing that some core technical performance parameters (including PassAcc) often exhibit limited between-group variation at elite level, even when tactical patterns differ [10, 12]. Accordingly, similar passing accuracy should not be interpreted as evidence of similar passing behaviour. In contrast, substantial between-league differences were observed in how passes were distributed and used to advance play. Previous empirical studies have demonstrated that passing decisions and distributions are strongly shaped by contextual constraints such as player–team positioning and situational information [28, 29], a conclusion also supported by systematic reviews highlighting the sensitivity of technical–tactical actions to match context (e.g., opponent strength, match status, and location) [30].

Among the leagues examined, J1 showed a clear emphasis on ball circulation and possession control. Teams in J1 recorded higher passing volume and greater use of forward, backward, lateral, and progressive passes than other leagues, alongside higher PassesPerMinute, PassesPerPossession, and Pass/DefensiveActions. These patterns are consistent with empirical studies of the J1 that emphasise the role of possession dynamics and passing-network structure in shaping team performance [31], without implying a fixed or deterministic national playing philosophy. By contrast, CSL, K1, SPL, and AGL exhibited higher Long Pass counts and Long Pass Proportions, indicating a shared tendency toward more vertical and direct offensive progression. In CSL, this pattern aligns with league-based evidence showing that passing behaviours and possession-related indicators vary systematically with contextual factors and team profiles [32].

K1 displayed a mixed passing identity that combined structured circulation with direct progression. Specifically, it showed greater use of back passes and progressive passes than CSL and AGL, while also exhibiting longer average pass lengths than J1. This configuration supports the interpretation that K1 integrates organised build-up play with rapid vertical transitions [6]. SPL and AGL demonstrated similar tendencies toward longer passing and reduced circulation compared with J1, consistent with styles prioritising territorial advancement over sustained possession. Empirical evidence linking higher ambient temperature and heat stress to changes in match-play characteristics, including passing and other technical actions, suggests that climate may contribute to these patterns as a contextual constraint [26, 33, 34]. Nevertheless, tactical preference remains the primary explanation, with environmental factors best viewed as complementary rather than determinative influences. Overall, passing behaviour provided the clearest empirical basis for distinguishing league-specific playing styles, with J1 emphasising structured circulation and the remaining leagues favoring more vertical and faster progression.

### Defensive outcome and Set-piece performance

Defensive and set-piece parameters showed relatively small differences across leagues, suggesting a shared level of defensive organisation and tactical standardisation across Asian competitions [1, 35, 36]. However, marked contrasts emerged in disciplinary and physical engagement profiles.

SPL, QSL, AGL, and CSL registered higher frequencies of fouls, yellow cards, free kicks, and penalties compared with J1 and K1, indicating a more physical, contact-oriented defensive style [6, 37, 38]. In contrast, J1 and K1 displayed higher aerial duel success, suggesting defensive systems that prioritise positioning, timing, and discipline over physical confrontation [39, 40]. K1 also achieved higher recoveries than AGL and CSL, aligning with its transitional defensive identity characterised by rapid ball regain [22]. Meanwhile, yellow card frequency was higher in K1 than in J1, which may reflect differences in refereeing tolerance, disciplinary standards, or game tempo [41–43]. AGL outperformed QSL in aerial duels, underscoring that even among leagues operating under broadly similar competitive and environmental conditions, variations in defensive emphasis and physicality persist [3, 44, 45].

### Limitations and future directions

Several limitations should be acknowledged. First, the present analysis did not incorporate player level physical or psychological states, nor did it explicitly model in match tactical adjustments across different phases of play. While the inclusion of contextual controls such as match result, location, team strength, and opponent strength reduces situational bias, the absence of player level and phase specific information may have obscured within league variability in tactical execution and adaptive behaviours.

Second, a number of potentially influential contextual factors such as match importance, coaching philosophy, squad rotation, and player nationality composition were not directly examined. These factors may interact with league level structures to shape technical and tactical behaviour and could partially account for residual differences observed between competitions.

Finally, although the longitudinal and multi league design strengthens the generalisability of the findings, the analysis was limited to six Asian professional leagues. Extending this framework to additional leagues, competitive tiers, and confederations would allow for a more comprehensive assessment of how cultural, organisational, and environmental contexts interact with tactical and technical performance. Future research would also benefit from integrating longitudinal player level data, advanced tracking or positional metrics, and phase of play classifications to better capture the dynamic and adaptive nature of elite football performance.

## CONCLUSIONS

This study compared technical and tactical match performance across six major Asian professional football leagues using a large, contextcontrolled dataset spanning six seasons. When controlling for match outcome, match location, and team and opponent strength, core offensive outcomes were largely similar across leagues, indicating a convergence in competitive effectiveness within Asian professional football.

Despite this convergence in outcomes, clear league-specific playing styles remained evident, particularly in passing structures and defensive–disciplinary profiles. Overall, the findings suggest that while Asian leagues are becoming increasingly similar in what they achieve in terms of match outcomes, they remain distinct in how tactical behaviours are expressed. These stylistic differences were most apparent in approaches to ball circulation, progression strategies, and defensive engagement, reflecting underlying differences in tactical emphasis across leagues. Future research should build on these findings by incorporating player tracking data, possession-value models, and analyses of micro-tactical sequences to better capture the dynamic and spatial mechanisms underpinning league-specific playing styles. Such approaches would allow for a more detailed understanding of how collective behaviours emerge from player interactions and contextual constraints. From a practical perspective, identifying stable stylistic differences across Asian professional football leagues may support coaches and performance analysts in opposition scouting, tactical preparation, and player recruitment, particularly in cross-league contexts such as international competition, coaching transitions, and player transfers.
